# The Clinical Effects of School Group Sandplay Therapy (SGST) on Anxiety/Depression, Somatic Symptoms, Social Immaturity, and Rule-Breaking Behavior in Children at Risk for ADHD

**DOI:** 10.3390/children12121592

**Published:** 2025-11-24

**Authors:** Hyo-Seong Han, You-Shin Yi, Myeong-Bok Lee, Heajin Shin, Youngil Lee, Chang Min Lee, Young Lim Lee, Myung Ho Lim

**Affiliations:** 1 Department of Psychology, Graduate School, Dankook University, Cheonan 31116, Republic of Korea; 2 Department of Anatomy, College of Medicine, Dankook University, Cheonan 31116, Republic of Korea; 3 Department of Neurology, College of Medicine, Dankook University, Cheonan 31116, Republic of Korea; 4 Department of Psychology and Psychotherapy, College of Health Science, Dankook University, Cheonan 31116, Republic of Korea

**Keywords:** children at risk of ADHD, school group sandplay therapy, SGST, sandplay therapy, depression, somatic symptoms, social immaturity, rule-breaking behavior

## Abstract

**Objectives**: Attention-Deficit/Hyperactivity Disorder (ADHD) in children is a developmental disorder that has been rapidly increasing worldwide. Its core symptoms, which include inattention, impulsivity, and hyperactivity, are often accompanied by emotional and behavioral problems such as depression and aggression. These factors can significantly impair a child’s development and functioning, making effective therapeutic intervention essential. This non-randomized controlled trial with parallel-group design examined the intervention effects of a 10-week school group sandplay therapy (SGST) program on internalizing and externalizing problems in children at risk for ADHD. **Methods**: This non-randomized controlled trial involved 101 fifth- and sixth-grade students (ages 11–12) from an elementary school in a mixed urban–rural area. Participants were divided into a control group (*n* = 47) and an intervention group (*n* = 54). The intervention group participated in a 10-session SGST program held once a week, while the control group received no intervention. The Korean Youth Self-Report (K-YSR) was used to evaluate the effects of the intervention, and a Repeated Measures ANOVA (RM-ANOVA) was conducted to analyze the program’s effectiveness. **Results**: The results revealed significant interaction effects between group and time in the SGST intervention group for several K-YSR subscales. The intervention group showed statistically significant differences in the areas of anxiety/depression (*p* < 0.04; η^2^ = 0.043), somatic symptoms (*p* < 0.04; η^2^ = 0.040), social immaturity (*p* < 0.01; η^2^ = 0.061), and rule-breaking behavior (*p* < 0.04; η^2^ = 0.044). **Conclusions**: SGST was found to be associated with improving not only internalizing problems like anxiety/depression, somatic symptoms in children at risk for ADHD but also externalizing problem like rule-breaking behavior and social problem like social immaturity. These findings demonstrate that school sand play therapy can be used as a non-pharmaceutical intervention for school-age children at risk of ADHD, and suggest that it can also be useful in an educational context.

## 1. Introduction

Attention-Deficit/Hyperactivity Disorder (ADHD) is one of the most common neurodevelopmental disorders in children, primarily characterized by symptoms of inattention, impulsivity, and hyperactivity [1]. In addition to these core symptoms, individuals with ADHD frequently face secondary challenges such as emotional dysregulation, impaired social skills, and academic struggles. Internalizing problems like anxiety, depression, and withdrawal, as well as externalizing problems like aggression and rule-breaking behavior, are often associated with ADHD. Therefore, a comprehensive and integrated therapeutic approach is essential [2].

According to a meta-analysis of 179 studies conducted across 26 countries, the average prevalence of ADHD among children and adolescents is 7.6% [3]. In the United States, the United Kingdom, Germany, and Australia, 5–10% of children and adolescents have been diagnosed with ADHD. In South Korea, the estimated prevalence of ADHD among school-aged children is approximately 5.9%, aligning with global average rates [4].

Additionally, recent statistics from the Korean Health Insurance Review and Assessment Service indicate a continued nationwide increase in the number of individuals diagnosed with ADHD. Notably, the number of adult ADHD cases rose by approximately 33%, from 18,000 in 2015 to 24,000 in 2019, highlighting the clinical significance of this disorder across the lifespan [5].

In this study, the ADHD risk group is defined as individuals who do not fully meet the diagnostic criteria for ADHD but exhibit some symptoms of inattention, hyperactivity, and impulsivity, accompanied by mild impairment in social or occupational functioning due to these symptoms. These individuals tend to struggle with interpersonal relationships and socio-emotional adaptation, may develop a negative self-concept, and exhibit characteristics of aggressive behavior [6]. Additionally, as reported by Orm et al. [7], individuals with ADHD are associated with psychological and emotional problems such as reduced self-control due to partial deficits in executive functioning, a higher risk of delinquency, potential substance abuse, depression, anxiety, somatic symptoms, and rule-breaking behavior. In this study, participants were classified as being at risk for ADHD if their total score on the ADHD Rating Scale-IV (ADHD RS-IV) was 16 or higher on the parent rating and 14 or higher on the teacher rating scale based on previous research [8]. While medication and behavioral therapy are the primary treatments for ADHD, various evidence-based group intervention programs have been developed in school settings, such as social skills training for children with ADHD [9].

Recently, various psychological approaches, such as play therapy and art therapy, have garnered attention as alternative or complementary treatments [10]. Among these, sandplay therapy has proven effective for children with limited verbal expression, allowing them to express their inner world and achieve emotional stability in a natural play environment [11].

According to prior research, School Group Sandplay Therapy (SGST) is effective in improving attention and reducing impulsivity in children with ADHD, and also shows significant results in enhancing self-regulation abilities and social skills. Wiersma et al. (2022) [12]’s systematic review and meta-analysis study (N = 1284) demonstrated that sandplay therapy maintains large effect sizes for internalizing symptoms (anxiety, depression, withdrawal), externalizing symptoms (aggression, behavioral problems), and ADHD symptoms (attention deficit/hyperactivity), providing the highest level of evidence for the effectiveness of sandplay therapy [13]. Recently, Chalfon and Ramos [14]’s randomized controlled trial showed that 12 weeks of individual sandplay therapy significantly reduced externalizing problems and aggressive behaviors in children with Oppositional Defiant Disorder (ODD) and Conduct Disorder (CD), demonstrating the effectiveness of sandplay therapy for behavioral problems co-occurring with ADHD.

SGST adapts traditional individual sandplay therapy into a group format, combining the benefits of individual therapy with the potential for improving social skills through peer interaction [15].

ADHD is a representative externalizing behavioral disorder that involves issues such as rule violations and aggression. Sandplay therapy has been reported to be effective not only for internalizing problems such as depression and anxiety but also for externalizing behaviors [15]. SGST enables nonverbal emotional expression, concrete and manipulable sensory activities, and the learning of social skills (such as peer interaction) within a structured and creative environment.

Indeed, previous studies have shown that group sandplay therapy can effectively reduce anxiety [16], improve social skills [13], and reduce aggression in children [17]. However, research on the effectiveness of SGST specifically for children at risk for ADHD remains very limited. Therefore, this study aimed to examine the effectiveness of SGST for children at risk for ADHD for the first time in East Asia, focusing on its impact on internalizing (Anxiety/Depression, Somatic Symptoms) and externalizing problems (Rule Breaking Behavior) through K-YSR.

## 2. Methods

### 2.1. Study Design

This study was conducted from May to July 2025 in a sandplay therapy room located within an elementary school in Cheonan, South Chungcheong Province, a mixed urban-rural area with a population of approximately 500,000. In addition, this research was designed as a non-randomized controlled trial. Application forms were distributed to each school, and participants from responding schools were assigned to groups based on classroom units within participating schools. The experimental group consisted of students from classrooms whose teachers voluntarily agreed to implement SGST, while the control group consisted of students from similar classrooms within the same schools that maintained regular classroom activities. No significant differences were found between the two groups regarding gender and age. Prior to implementation, a therapy plan was developed with the cooperation of school teachers and consultation from three experts. Program attendance and fidelity were managed using a checklist, and groups were composed of mixed genders. The control group did not receive any therapeutic intervention, or any psychosocial support during the research period. The intervention group participated in the SGST program once a week for 40 minutes over a total of 10 sessions, while the control group did not receive any intervention. All participants were evaluated before and after the intervention. The SGST sessions were conducted by a therapist with over 10 years of experience in sandplay therapy, under the clinical supervision of a child psychiatrist.

### 2.2. Participants

**Figure 1:**
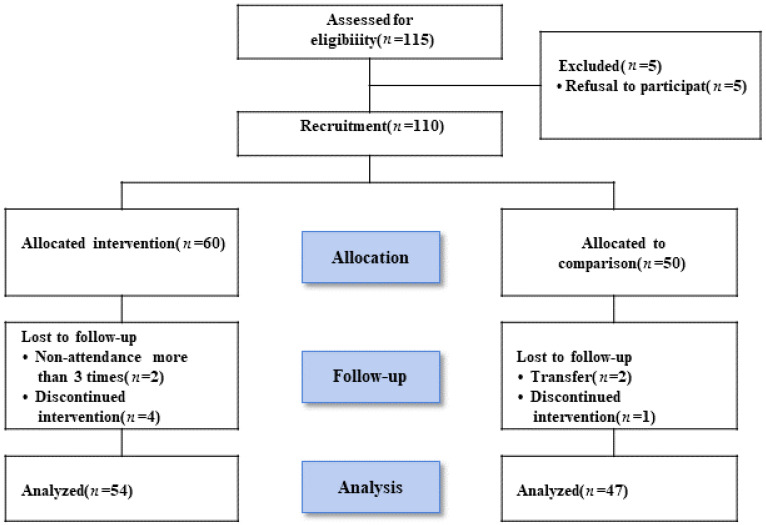
Flow diagram of participants.

A survey related to ADHD was conducted using the ADHD Rating Scale-IV (ADHD RS-IV) among fifth- and sixth-grade students at an elementary school in Cheonam city. Of the 115 students who showed symptoms of inattention, hyperactivity, and impulsivity, five declined to participate. Ultimately, 110 students who agreed to participate were selected as study subjects after obtaining parental consent. Among them, 60 were assigned to the intervention group and 50 to the control group. After excluding two students due to insufficient attendance (fewer than three sessions) and four for dropping out, 54 participants remained in the intervention group. In the control group, two students who transferred schools and one who dropped out were excluded, leaving 47 participants in the final analysis (Figure 1). None of the participants were receiving medication treatment or had comorbid severe neuropsychiatric disorders.

### 2.3. SGST Program

SGST was conducted once a week over a total of ten sessions (40 min/session). Small groups of 3 to 4 children of similar age were formed, and each child was provided with an individual sand tray at individual classroom. The figures and materials used during the sessions were shared among group members. After completing their work, participants were given time to present and reflect on each other’s creations. The therapist facilitated interaction among group members and encouraged the children to freely express their emotions. The program was adapted and refined based on Boik & Goodwin’s Sandplay Therapy [18], Kalff’s Sandplay Therapy [19]. The SGST program developed by Kwak et al. [20], consist of departure, emotional self-awareness, conflict and struggle, emotional expression of family/friends/school, self-understanding and acceptance, and integration (Table 1).

### 2.4. Measures

#### 2.4.1. ADHD Rating Scale-IV: ADHD RS-IV

In this study, the ADHD RS-IV was used to assess children’s ADHD tendencies. Developed by DuPaul [21], this scale serves as a behavioral assessment tool for school-aged children. The Korean version was validated by So et al. [22] standardized by Kim et al. [8] for children aged 7 to 12. The ADHD RS-IV consists of 18 items based on the DSM-IV [23] diagnostic criteria, with odd-numbered items addressing inattention and even-numbered items focusing on hyperactivity-impulsivity. Each item is rated on a 4-point Likert scale ranging from 0 to 3. In this study, the internal consistency reliability (Cronbach’s α) was 0.94 for the parent rating and 0.96 for the teacher rating.

#### 2.4.2. Youth Self-Report (YSR)

To examine the effectiveness of SGST and particularly to investigate its impact on internalizing, externalizing, and problem behaviors, we set the anxiety/depression subscale of the K-YSR as the primary outcome measure for this study. Secondary outcome measures included the somatic symptoms, social immaturity, and rule-breaking behavior subscales of the K-YSR. This study used the Youth Self-Report (YSR) for adolescents (11–18 years old), which was developed by Achenbach & Rescorla [24] and standardized in Korean by Oh & Kim [25]. The YSR consists of 112 items and includes eight syndrome scales: Anxiety/Depression, Withdrawal/Depression, Somatic Symptoms, Social Immaturity, Cognitive Issues, Attention Problems, Rule-Breaking Behavior, and Aggressive Behavior. Additionally, it provides scores on three higher-order scales: internalizing problems, externalizing problems, and total problems. The reliability of the Korean version of the YSR ranged from 0.59 to 0.93. Also in this study, the internal consistency reliability (Cronbach’s α) for each subscale was 0.87~0.90.

### 2.5. Statistical Analysis

Data analysis was performed using IBM SPSS Statistics 28.0. RM-ANOVA was conducted with group (intervention, control) and time (pre-test, post-test) to evaluate the effects of the SGST intervention.

### 2.6. Ethical Considerations and Informed Consent

This study was conducted in accordance with the Declaration of Helsinki and received approval from the Institutional Review Board of Dankook University. Written informed consent was obtained from all participants and their legal guardians after explaining the purpose and procedures of the study. Participants were also informed that they could withdraw from the study at any time without any penalty.

## 3. Results

### 3.1. Demographic Characteristics

We described the demographic characteristics of the intervention group (*n* =54) and the control group (*n* = 47) in Table 2.

The mean age of the control group was 11.38 ± 0.72 years, while that of the intervention group was 11.39 ± 0.67 years, indicating no statistically significant difference between the two groups (t = 0.06, *p* = 0.952).

Regarding gender distribution, the control group consisted of 17 males (36.2%) and 30 females (63.8%), while the intervention group included 22 males (40.7%) and 32 females (59.3%). There was no statistically significant difference in gender distribution between the two groups (χ^2^ = 0.64, *p* = 0.22).

### 3.2. Analysis of the SGST Effects

The results of the RM-ANOVA for the YSR subscale scores of the intervention and control groups are summarized in Table 3.

The RM-ANOVA results revealed significant group × time interaction effects across four YSR subscales. For the anxiety/depression domain, significant main effects of time (F = 7.86, *p* < 0.01) and group × time interaction (F = 4.46, *p* < 0.04) were found. Significant group × time interaction effects were found for somatic complaints, social immaturity, and rule-breaking behavior domains (F = 4.16, 6.89, 4.54, respectively, all *p* < 0.05).

The SGST program showed significant effects in the domains of Anxiety/Depression, Somatic Symptoms, Social Immaturity, and Rule-Breaking Behavior, these results indicate that the SGST program reduces physical complaints, increases emotional stability, enhances social maturity, and decreases oppositional defiant behavior.

## 4. Discussion

This study examined the effectiveness of SGST for children at risk for ADHD. The results demonstrated that SGST was associated with improving internalizing problems (anxiety/depression and somatic symptoms), externalizing problems (rule-breaking behavior), and social difficulties (social immaturity) among children at risk for ADHD.

First, in the internalization (Anxiety/Depression, Somatic Symptom) domain, a significant group × time interaction effect was observed following the SGST intervention. This finding aligns with previous research. Rousseau et al. (2009) [26] reported that sandplay therapy reduced emotional difficulties such as anxiety and depression in 105 children from multicultural families. Similarly, conducting group sandplay therapy for children experiencing emotional and behavioral difficulties resulted in significant reductions in Anxiety/Depression, social issues, and aggression as measured by the Strengths and Difficulties Questionnaire (SDQ). These effects may be attributed to the therapeutic mechanisms of SGST, which facilitate emotional expression through nonverbal communication using the tactile elements of sand and figures [27]. In group sandplay settings, peer interactions could also have contributed to alleviating Anxiety/Depression by providing emotional support and fostering a sense of connection. Lee et al. [28] found that sandplay therapy significantly improved symptoms of depression and anxiety in a study involving juvenile delinquents, supporting the findings of this study. Also, Roubenzadeh & Abedin [29] reported improvements in both negative emotions and somatic symptoms through short-term sandplay therapy for adolescents dealing with the loss of close family members. Kim et al. [30] and Cao et al. [31] found that sandplay therapy was effective in reducing somatic symptoms and negative emotions in middle school students with PTSD. Given that children often express symptoms of depression, anxiety, and fear through somatic symptoms [20], the results of this study also suggest that group sandplay therapy had a positive effect on somatized emotional difficulties. Sandplay served as a safe medium for children with limited verbal expression to convey their inner world, likely helping to mitigate the transformation of repressed emotions into somatic symptoms.

Second, a significant group × time interaction effect was also observed in the Externalization (Rule-Breaking Behavior) domain, suggesting that SGST contributed to the emotional stability of children with ADHD [26]. Similar findings have been reported by Kwak et al. [20] and Wang [32], who found that group sandplay therapy for children with behavioral problems led to significant improvements in externalizing behaviors, including noncompliance with parental expectations, rule-breaking behavior, and aggression. Notably, despite targeting children at risk for ADHD, no significant effects were observed on cognitive/attention-related subscales, while relatively large effect sizes were seen in rule-breaking behavior. This suggests that SGST may be effective for hyperactivity/impulsivity symptoms among the core symptoms of ADHD, but may be limited in addressing attention deficit symptoms.

Third, the strongest group × time interaction effect was observed in the Social problem (Social Immaturity) domain (F = 6.89, *p* < 0.01). Children diagnosed with ADHD often struggle with peer relationships due to challenges with social cue recognition, impulsivity, and self-regulation [33]. In this study, SGST served as an effective intervention for addressing such social difficulties. The group format of sandplay therapy provides a structured and safe environment for children to interact with their peers, facilitating the learning of social norms and the development of appropriate interpersonal skills [33]. Through activities in the sand tray, such as taking turns, sharing figures, and respecting each other’s creations, children were able to naturally acquire essential social interaction skills. These findings align with numerous previous studies. Kim et al. [30] and Zheng & Hu [34] reported that adolescents from divorced families who participated in sandplay therapy exhibited significant improvements in interpersonal trust. They noted that the safe therapeutic relationships formed during the sandplay process, coupled with positive experiences within the group, contributed to restoring trust in interpersonal relationships.

Kim et al. [30] and Kazemi et al. [35] confirmed that sandplay therapy significantly improved social skills and adaptability in children with separation anxiety disorder. They found that engaging in sandplay activities improved children’s self-expression and alleviated anxiety about social interactions, positively influencing their peer relationships.

The group format of SGST offers advantages over individual sandplay therapy, as it allows participants to directly experience peer interactions and receive feedback. The significant improvement observed in the Social Immaturity domain in this study can be interpreted as a result of children at risk for ADHD receiving immediate feedback on their behavior during the group sandplay process, as well as having opportunities to observe and imitate the adaptive behaviors demonstrated by their peers. These findings suggest that SGST is an effective intervention for enhancing social skills in children at risk for ADHD.

Additionally, the observed improvements in cognitive issues and attention issues indicate that sandplay activities contributed to better attention in children at risk for ADHD. Creative activities involving sand and figures effectively stimulate children’s interest and help them focus naturally [36].

However, this study has some limitations. First, the reliance on self-report measures limited the scope of objective behavioral assessments. Future studies should incorporate comprehensive research that includes not only student self-assessments but also parent and teacher evaluations to enhance inter-rater reliability. Second, no follow-up assessment was conducted after the 10-week sandplay intervention, so the long-term effects of SGST could not be examined. Third, since SGST was implemented in a single elementary school located in a mixed urban-rural area, there are limitations in generalizing the program’s effectiveness. Fourth, furthermore, since school teachers assigned participants to groups, this study is a non-randomized controlled parallel trial of experimental research, which may be affected by selection bias and school cluster effects. In this study, since we classified the ADHD risk group based on K-ADHD RS scale scores, there may be a risk of misclassification compared to clinical diagnosis. Future research should include randomized controlled trials that control for gender, age, economic status, selection bias, and cluster effects. We anticipate studies that include regional schools nationwide as participants, utilize more diverse assessment tools, and conduct long-term follow-up evaluations. Investigating the comparative effectiveness or combined effects of SGST versus other treatments, such as medication or behavioral therapy, would also be beneficial.

Based on these results, SSGT in school settings can help children regulate their emotions, reduce somatic complaints, better respect with school rules, and improve their ability to coexist with peers.

## 5. Conclusions

SGST showed statistically significant differences on internalizing and externalizing problems as well as social skills improvement in children at risk for ADHD. Statistically significant association were particularly observed in anxiety/depression, somatic complaints, social immaturity, and rule-breaking behavior. These findings suggest that SGST could potentially serve as a useful psychotherapeutic intervention for children at risk for ADHD, though the results should be interpreted cautiously given the methodological limitations of this study. Future studies should aim to validate the effects of SGST through long-term follow-up research and large-scale investigations while developing SGST protocols tailored to the specific needs of children at risk for ADHD.

## Figures and Tables

**Table 1:** SGST program highlights.

Directives	Session	Sandplay Therapy Activities
Departure	1	Feel the texture of the sand and express your thoughts and feelings.
Emotional self-awareness	2	Close your eyes. Touch the sand and express the emotions that arise from its texture in the sand tray.
	3	Express the emotions you feel when recalling past memories.
Conflict and struggle	4	Express the uncomfortable feelings and emotions you are currently experiencing.
	5	Recall and express an image of fighting against difficult situations.
Family. friends, and school	6	Express your feelings while thinking about your family.
	7	Express your thoughts and feelings about friends and school.
Self understanding and acceptance	8	Express yourself in the sand tray.
	9	Recall the sandwork you’ve created thus far and express your feelings.
Integration	10	Imagine and express a new version of yourself.

**Table 2:** Demographic characteristics of the intervention group (*n* = 54) and control group (*n* = 47).

Variables	Intervention Group (*n* = 54)	Control Group (*n* = 47)	Test Statistic	*p* Value
Age ^a^	11.39 ± 0.67	11.38 ± 0.72	*t* = 0.06	0.952
Sex ^b^			x^2^ = 0.64	0.22
Male	22 (40.7%)	17 (36.2%)		
Female	32 (59.3%)	30 (63.8%)		

^a^ Age was analyzed using an independent *t*-test, and ^b^ sex was analyzed using a chi-square test; Values in parentheses indicate percentages (%).

**Table 3:** Pre- and post-test differences between intervention (N = 54) and control groups (N = 47) using RM-ANOVA (*n* = 101). * *p* < 0.05, ** *p* < 0.01.

Variables	Group	Mean ± SD		Group F(P)	Time F(P)	Group × Time F(P)	η^2^
		Pre	Post				
Anxiety/Depression	Control	55.15 ± 7.65	55.02 ± 7.85	2.45 (0.12)	7.86 ** (0.01)	4.46 (0.04)	0.043
	Intervention	59.63 ± 10.91	55.80 ± 10.67				
Withdrawal/Depression	Control	53.89 ± 5.20	53.72 ± 5.19	8.48 ** (0.004)	5.53 * (0.02)	3.56 (0.06)	0.035
	Intervention	59.56 ± 10.48	56.76 ± 9.92				
Somatic Symptoms	Control	53.15 ± 5.37	53.87 ± 5.51	2.95 (0.89)	3.65 (0.06)	4.16 * (0.04)	0.04
	Intervention	56.35 ± 6.54	54.30 ± 7.36				
Social Immaturity	Control	53.89 ± 6.28	55.21 ± 6.96	4.85 (0.03)	4.40 (0.04)	6.89 (0.01)	0.061
	Intervention	58.63 ± 8.59	56.28 ± 7.91				
Cognitive Issues	Control	55.09 ± 6.68	54.62 ± 7.62	7.82 (0.01)	1.27 (0.27)	0.39 (0.53)	0.004
	Intervention	59.93 ± 10.03	58.4 ± 9.93				
Attention Problems	Control	52.81 ± 5.23	52.49 ± 4.69	4.96 (0.03)	4.95 (0.03)	2.764 (0.1)	0.027
	Intervention	56.76 ± 9.49	53.69 ± 7.55				
Rule-Breaking Behavior	Control	53.17 ± 4.50	54.64 ± 6.04	1.59 (0.21)	1.01 (0.32)	4.54 (0.04)	0.044
	Intervention	55.78 ± 7.30	54.85 ± 6.67				
Aggressive Behavior	Control	55.49 ± 6.28	55.45 ± 7.08	0.63 (0.43)	5.48 (0.02)	3.24 (0.07)	0.032
	Intervention	57.76 ± 8.39	55.26 ± 7.61				

## Data Availability

The datasets generated during and/or analyzed during the current study are available from the corresponding author on reasonable request.
